# Altered Topological Properties of Grey Matter Structural Covariance Networks in Complete Thoracic Spinal Cord Injury Patients: A Graph Theoretical Network Analysis

**DOI:** 10.1155/2021/8815144

**Published:** 2021-02-01

**Authors:** Wen-Li Wang, Yu-Lin Li, Mou-Xiong Zheng, Xu-Yun Hua, Jia-Jia Wu, Fei-Fei Yang, Nan Yang, Xia He, Li-Juan Ao, Jian-Guang Xu

**Affiliations:** ^1^School of Rehabilitation, Kunming Medical University, Yunnan, China; ^2^Department of Rehabilitation Medicine Science, Shanghai University of Traditional Chinese Medicine, Shanghai, China; ^3^Department of Traumatology and Orthopedics, Yueyang Hospital of Integrated Traditional Chinese and Western Medicine, Shanghai University of Traditional Chinese Medicine, Shanghai, China; ^4^Department of Rehabilitation Medicine, Yueyang Hospital of Integrated Traditional Chinese and Western Medicine, Shanghai University of Traditional Chinese Medicine, Shanghai, China; ^5^The Second Affiliated Hospital of Kunming Medical University, Yunnan, China

## Abstract

**Purpose:**

This study is aimed at investigating brain structural changes and structural network properties in complete spinal cord injury (SCI) patients, as well as their relationship with clinical variables.

**Materials and Methods:**

Structural MRI of brain was acquired in 24 complete thoracic SCI patients (38.50 ± 11.19 years, 22 males) within the first postinjury year, while 26 age- and gender-matched healthy participants (38.38 ± 10.63 years, 24 males) were enrolled as control. The voxel-based morphometry (VBM) approach and graph theoretical network analysis based on cross-subject grey matter volume- (GMV-) based structural covariance networks (SCNs) were conducted to investigate the impact of SCI on brain structure. Partial correlation analysis was performed to explore the relationship between the GMV of structurally changed brain regions and SCI patients' clinical variables, including injury duration, injury level, Visual Analog Scale (VAS), American Spinal Injury Association Impairment Scale (AIS), International Classification of Functioning, Disability and Health (ICF) scale, Self-rating Depression Scale (SDS), and Self-rating Anxiety Scale (SAS), after removing the effects of age and gender.

**Results:**

Compared with healthy controls, SCI patients showed higher SDS score (*t* = 4.392 and *p* < 0.001). In the VBM analysis, significant GMV reduction was found in the left middle frontal cortex, right superior orbital frontal cortex (OFC), and left inferior OFC. No significant difference was found in global network properties between SCI patients and healthy controls. In the regional network properties, significantly higher betweenness centrality (*BC*) was noted in the right anterior cingulum cortex (ACC) and left inferior OFC and higher nodal degree and efficiency in bilateral middle OFCs, while decreased *BC* was noted in the right putamen in SCI patients. Only negative correlation was found between GMV of right middle OFC and SDS score in SCI patients (*r* = −0.503 and *p* = 0.017), while no significant correlation between other abnormal brain regions and any of the clinical variables (all *p* > 0.05).

**Conclusions:**

SCI patients would experience depressive and/or anxious feelings at the early stage. Their GMV reduction mainly involved psychology-cognition related rather than sensorimotor brain regions. The efficiency of regional information transmission in psychology-cognition regions increased. Greater GMV reduction in psychology region was related with more severe depressive feelings. Therefore, early neuropsychological intervention is suggested to prevent psychological and cognitive dysfunction as well as irreversible brain structure damage.

## 1. Introduction

The global incidence of spinal cord injury (SCI) is 250,000 to 500,000 every year, and the major etiology is trauma, including road traffic injuries, falls, and violence, which mostly occurs in males [1, 2]. SCI is a kind of permanent neurological disorders characterized by a wide range of dysfunctions in motor, sensory, and autonomic nervous systems below the level of injury. It not only reduces patients' self-efficacy and the quality of life (QoL), but also leads to structural and functional brain reorganization.

The underlying pathophysiological process of SCI consists of primary and secondary phases [3]. The primary phase is the initial injury suffered upon impact, leading the direct mechanical injury for some neurons [4]. Because most of the posttraumatic neurochemical mediators of secondary injury may act as neurodestructive compounds, the secondary phase mainly referred to the progressive neurodegeneration, including autophagy and apoptosis at sites that are distant to the vicinity of primary injury [5–8]. These morphological changes may play an important role in the pathophysiology of the brain cortex and could be directly examined by immunohistochemical techniques [9]. However, it is impossible to apply immunohistochemical techniques in human beings, which makes it difficult to evaluate structural changes in human's brain following SCI.

Structural magnetic resonance imaging (MRI) is a noninvasive technique that allows direct access to the structural changes of the human brain. Voxel-based morphometry (VBM) analysis is a valid and spatially unbiased automated method that quantifies structural changes in white and gray matter volume (WMV and GMV) in living human brain [10]. Both adaptive and maladaptive reorganization may occur following SCI [11]. Previous studies revealed that SCI reduced the GMV in multiple brain regions, including the primary motor cortex (M1) [12, 13], primary sensory cortex (S1) [14, 15], supplemental motor area (SMA) [16], inferior frontal cortex [17], bilateral orbital frontal cortices (OFCs), dorsal anterior cingulate cortex (ACC), bilateral anterior insular cortices, and right superior temporal gyrus [14, 18]. But mere morphometric analysis may be inadequate to uncover the structural reorganization mechanisms [19]. In the present study, we reported a covariant change of structural network in a group of subjects, which may provide a new perspective for this issue.

Structural covariance network (SCN) constructed from cross-subject morphometric correlations is another useful method to explore the structural reorganization mechanisms in living human brain. Structural covariances are defined as the interindividual differences in the morphology of one brain region covary with other brain regions. SCNs are constructed generally with one of three experimental approaches including seed-based analysis, principal component analysis, and graph theoretical network analysis [20]. Graph theoretical network analysis is a useful analytic tool in studying brain structural network [21]. The topological properties of SCNs including global and regional covariance parameters could be explored accordingly [22]. The graph theoretical network analysis of SCNs has been widely used in neurodegenerative diseases (e.g., Parkinson disease [23, 24] and Alzheimer disease [25]) and psychological diseases (e.g., obsessive-compulsive disorder [26, 27], schizophrenia [28] and major depression [29]). The brain morphometric and topological properties in these diseases were found rearranged, providing a fundamental for further improvement of clinical diagnose and specific treatments. How the human brain reacts to the disconnection of motor efferent and sensory afferent after SCI has been attracting more and more attention. Graph theoretical network analysis has been applied in resting-state functional MRI (rs-fMRI) [26, 27] and electroencephalography (EEG) studies [30] in SCI patients. However, to our knowledge, previous studies more focused on functional network changes [31–33]. The alterations of the topological properties of SCNs in brain at the early stage following SCI are largely unknown.

In the present study, we aimed to investigate the GMV changes with VBM analysis and to estimate topological properties of GMV-based SCNs using graph theoretical network analysis by characterizing and comparing the graph theoretical metrics between SCI patients and healthy controls. The potential correlation between GMV of structurally changed brain regions and clinical variables will also be explored. Combination of structural and structural covariance network information may provide more comprehensive knowledge about how brain reorganized after SCI. It may also be helpful to modify current strategy of rehabilitation sessions by providing more accurate and specific trainings for SCI patients at early stage to prevent irreversible brain structural changes.

## 2. Materials and Methods

### 2.1. Participants

From November 2018 to December 2019, we recruited 24 patients with complete thoracic SCI (38.50 ± 11.19 years, 22 males) from the Department of Rehabilitation, the Second Affiliated Hospital of Kunming Medical University. And 26 healthy controls (38.38 ± 10.63 years, 24 males) were enrolled in this study. SCI patients underwent cross-sectional evaluation of clinical assessments and MR scanning. Age- and sex-matched healthy controls were recruited from the community. Inclusion criteria of SCI patients are as follows: (1) age between 20 and 60 years old, no gender limitation; (2) complete SCI (AIS score = A); (3) traumatic SCI at thoracic level; (4) with a duration of less than 1 year from injury. Participants who had a history of traumatic brain injury, brain deformity, mental illness, epilepsy, cognitive impairment, or contraindications of MR scanning were excluded.

The study protocol was approved by the Ethics Committee of the Second Affiliated Hospital of Kunming Medical University, Yunnan, China (No.: FEY-BG-39-2.0). The purposes and procedures of this study were explained to all of the participants at recruitment. All participants provided written informed consent before enrolment.

### 2.2. Clinical Assessments

#### 2.2.1. American Spinal Injury Association Impairment Scale (AIS)

AIS (https://asia-spinalinjury.org/) is usually used to define severity of SCI injury and the level of neurological impairment. It contains three components including myotomal-based motor assessment, dermatomal-based sensory assessment, and anorectal assessment [34].

#### 2.2.2. Psychological Impact

Self-rating Depression Scale (SDS) and Self-rating Anxiety Scale (SAS) were used for quantifying depressive and anxious status, respectively [35, 36]. Both scales are 20-item scales associated with affective and somatic symptoms (10 negative and 10 positive experience items in SDS; 15 negative and 5 positive experience items in SAS), with a normalized total score ranged from 25 to 100. The score of SDS/SAS ranges 50-59, 60-69, and ≥70 indicated mild-moderate, moderate-severe, and severe depression/anxiety, respectively [37, 38].

#### 2.2.3. Pain

Visual analog scale (VAS) is a 10 cm line with the endpoints labeled “no pain” (score of 0) and “worst possible pain” (score of 10), with a higher score of VAS indicates greater pain [39]. All of SCI patients were asked to mark the VAS according to their pain intensity, and the score was recorded by measuring the distance from 0 to the mark (cm).

#### 2.2.4. Functional Impairment

International Classification of Functioning, Disability and Health (ICF) is a framework for the description, assessment, and classification of ability and disability in the areas of *body functions* and *body structures*, *activities*, *participation*, *personal factors*, and *environmental factors* [40]. The “Spinal Cord Injury Long-Term Brief Cord Set” was used to describe most relevant functionality in SCI patients. It includes 33 items, and each item has 7 categories. A higher score indicates greater impairment of functionality [41].

### 2.3. MRI Acquisition

The MR images were collected by a 3T Philips MRI system with an 8-channel phase-array head coil. A three-dimensional magnetization-prepared rapid gradient echo sequence (MPRAGE) was applied to obtain high-resolution 3D structural T1-weighted images in sagittal orientation. The imaging parameters were as follows: repetition time (TR) = 7.87 ms, echo time (TE) = 3.83 ms, flip angle (FA) = 15°, field of view (FOV) = 256 × 256 mm^2^, matrix size = 256 × 256, and slice thickness = 1 mm, resulting an isotropic voxel size of 1 × 1 × 1 mm^3^.

### 2.4. Structural Data Preprocessing and Whole-Brain VBM Analysis

The VBM analysis was performed using the VBM8 toolbox in the Statistical Parametric Mapping software (SPM8, Wellcome Department of Cognitive Neurology, UCL, http://www.fil.ion.ucl.ac.uk/spm/) implemented in the Matlab environment (2014a, The MathWorks Inc.). After converting DICOM files into NIfTI images, all T1-weighted images were visually checked and poor-quality images with apparent artifacts would be excluded if any. And then, the anatomic image of each participant was reoriented so that the origin approximated the anterior commissure and the orientation approximated Montreal Neurological Institute (MNI) space. The T1-weighted structural images were segmented into grey matter (GM), white matter (WM), and cerebrospinal fluid (CSF) components. The GM images were spatially normalized into the standard MNI space by applying the Diffeomorphic Anatomical Registration using Exponentiated Lie (DARTEL) algorithm. The normalized GM component was modulated by nonlinear transform only and then smoothed with Gaussian kernel of full width at half-maximum (FWHM) (8 mm). The GM, WM, and CSF volumes were obtained separately, based on the segmented images. The total intracranial volume (TIV) was calculated as the sum of all the GM, WM, and CSF volumes.

### 2.5. Structural Covariance Network Construction

**Figure 1:**
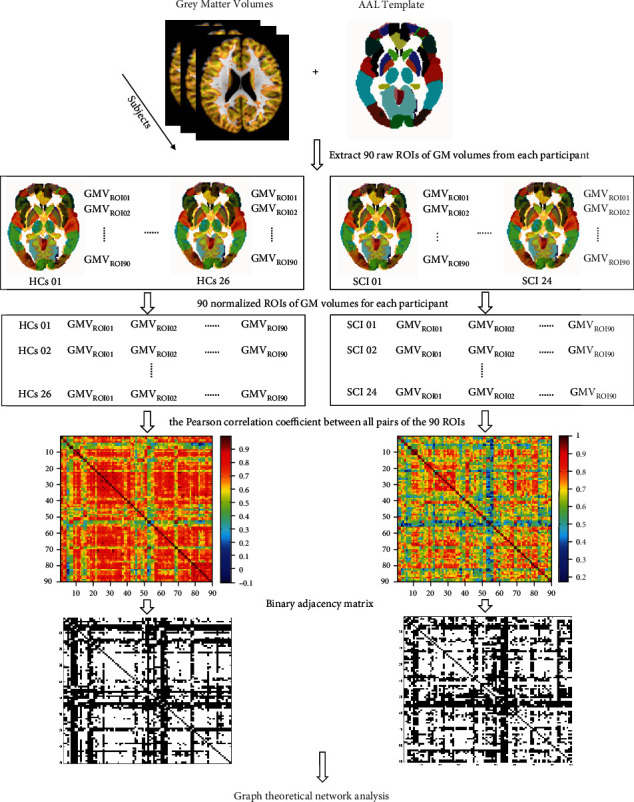
The diagram of structural covariance network construction and analysis. AAL: anatomical automatic labeling; GMV: grey matter volume: ROI: region of interest; HCs: healthy controls; SCI: spinal cord injury.

Pearson correlation was used to compute structural covariance based on GMV. SCN construction and network property analysis were processed with Brain Connectivity Toolbox (BCT, version 2017-15-01, http://www.brain-connectivity-toolbox.net/). The process of SCN construction is shown in Figure 1.

Regional parcels for GMV were extracted based on the Automatic Anatomic Labeling (AAL) atlas, including 90 region of interests (ROIs) [42]. Each of all the 90 AAL brain regions was considered as a node of a network. The effects of age and gender were regressed out using generalized linear model before network construction. The edge of the network was defined as the Pearson correlation coefficient of the normalized GMV between all pairs of the 90 regions across the participants in the same group [43]. Thus, a single network was generated for each group. To ensure that all the resultant networks had the same number of edges, the range of sparsity was set for each correlation matrix, which allowed prominent small-world properties in brain networks to be observed. According to the literature, we restricted each absolute correlation matrix to a specific range of sparsity (0.05-0.5) at an interval of 0.01 [44, 45]. Through this thresholding procedure, we obtained a set of undirected and unweighted binarized networks. Graph theoretical network analysis was then used to investigate the topological properties of whole-brain structural networks in SCI patients and healthy controls.

#### 2.5.1. Global Network Analysis

Topological properties of global network including clustering coefficient, characteristic path length, small-worldness index, global efficiency and local efficiency were used to characterize the global topological organization of SCNs (Details in the Supplemental Materials).

#### 2.5.2. Regional Network Analysis

Three topological properties of regional network including degree (*D*), nodal efficiency (*E*_nod_), and betweenness centrality (*BC*) were used to demonstrate the nodal characteristics of the SCNs [46]. Nodal degree *D* is defined as the sum of all direct connections between a given node and other nodes, and the index *D* is used to measure the importance of a node. Nodal efficiency (*E*_nod_) is the mean reciprocal of the shortest path length between a given node and all the others, and *E*_nod_ is an evaluation for regional connectivity. The *BC* is defined as the fraction of all shortest paths in the network that passed through a given node, and *BC* is used to find important anatomical or functional connections.

### 2.6. Statistical Analysis

#### 2.6.1. Baseline Characteristics and Clinical Assessment

The baseline characteristics and clinical assessment data were analyzed using the statistical package IBM SPSS Statistics V21 (SPSS Inc., Chicago, IL, USA). Data were assessed for normality using the Kolmogorov-Smirnov test. Mean (SD, standard deviation) was used to describe normally distributed data, and intergroup differences were analyzed using two-sample *t*-test.

#### 2.6.2. Voxel-Based Morphometry Analysis

GMV differences between SCI patients and healthy controls were compared using voxel-wise two-sample *t*-test with the function of “second level model” in SPM8. The multiple comparison was corrected by voxel-wise family-wise error (FWE) method with significance level of *p* < 0.05. The results of VBM statistical analysis were visualized using the MRIcroGL (Version 11, https://www.mccauslandcenter.sc.edu/mricrogl/) and GraphPad (Inc. Prism Version 7, US, https://www.graphpad.com/).

#### 2.6.3. Graph Theoretical Network Analysis

Intergroup difference of structural covariance network topological properties was analyzed by using nonparametric permutation test with 5000 repetitions. In each permutation cycle, the GMV of ROI for each participant was randomly assigned to either SCI or healthy control group to form two new groups with the same size in each group as the original ones. Then, the randomized, undirected, and unweighted binarized network of each group was constructed based on the new dataset. The network properties in each permutation cycle were calculated across all sparsity thresholds, resulting a corresponding area under the curve (AUC) over the sparsity range of both groups. Intergroup differences of 5000 AUCs constituted permutation distribution under null hypothesis; the *p* value was obtained according to the position of the actual intergroup difference of AUC of network properties at the permutation distribution. The significance level for global network analysis was set at *p* < 0.05. And the significance level for regional network analysis was set at *p* < 0.05 after FDR correction for multiple comparisons.

### 2.7. Partial Correlation Analysis

Partial correlation analysis using the statistical package IBM SPSS Statistics V21 (SPSS Inc., Chicago, IL, USA) was conducted at ROI-wise to explore potential correlation between the GMV and injury duration, injury level, VAS, AIS, ICF scale, SDS, and SAS, respectively, after removing the effects of age and gender. The selection of ROIs was based on the brain regions presented intergroup differences both in VBM and network analyses. The results of partial correlation analysis were also visualized using the statistical package IBM SPSS Statistics V21 (SPSS Inc., Chicago, IL, USA).

## 3. Results

### 3.1. Demographic Characteristics and Clinical Details

The interval from injury to administration to our clinic was 5.58 ± 3.15 (mean ± SD) months (ranged from 1 to 11 months). The motor and sensory score were 50.46 ± 4.80 and 126.19 ± 23.38, respectively. The VAS score of SCI patients was 3.96 ± 2.46 (mean ± SD). Detailed information of demographic characteristics and clinical variables of SCI patients are listed in Table 1. The intergroup differences of demographic characteristics and clinical variables are listed in Table 2. There was no intergroup difference regarding age (*t* = 0.037 and *p* = 0.970) or gender (*χ*^2^ = 0.007 and *p* = 0.933) or SAS score (*t* = 0.988 and *p* = 0.328). The SDS score of SCI patients was higher than healthy controls (*t* = 4.392 and *p* < 0.001). In the preprocessing procedure, all the MR images were qualified and no participant was excluded from subsequent analysis.

### 3.2. Reduction of GMV in SCI Patients

**Figure 2:**
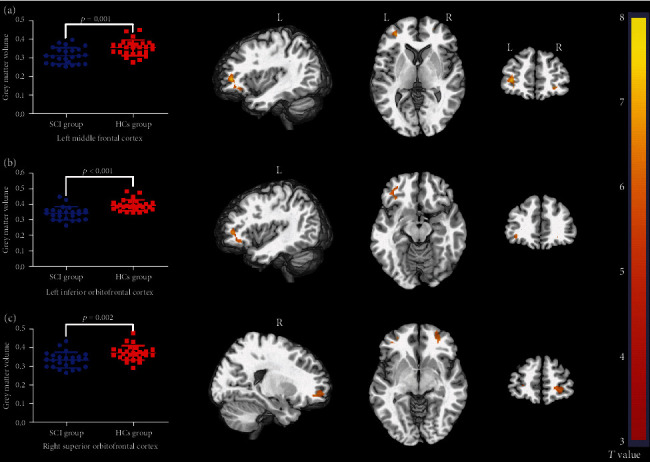
The results of voxel-based morphometry (VBM) analysis. Regions in (a) left middle frontal cortex, (b) left inferior orbitofrontal cortex, and (c) right superior orbitofrontal cortex showed significantly lower grey matter volume (GMV) in spinal cord injury (SCI) patients compared with healthy controls (HCs) (voxel-wise FWE correction with *p* < 0.05). SCI: spinal cord injury; HCs: healthy controls; L: left; R: right.

Voxel-wise two-sample *t*-test was thresholded at voxel-wise FWE correction with *p* < 0.05. The results of VBM analysis revealed that compared with healthy controls, SCI patients showed significant GMV decrease in the left middle frontal cortex, right superior orbital frontal cortex (OFC), and left inferior OFC (Figure 2). No brain region showed greater GMV in SCI patients than the healthy controls. The cluster size, peak *T* value, and peak MNI coordinates of regions with decreased GMV are listed in Table 3.

### 3.3. Differences of Topological Properties of SCNs between SCI Patients and Healthy Controls

There was no significant difference in topological properties of global network between SCI patients and healthy controls (all *p* > 0.05) (Supplementary Materials, Table S1, Figure S1).

Intergroup differences of the regional network parameters are listed in Table 4. Compared with healthy controls, SCI patients showed significantly higher *BC* in the right ACC and left inferior OFC and higher degree and efficiency in bilateral middle OFCs. *BC* in the right putamen in SCI patients was significantly lower than that in healthy controls.

### 3.4. Relationship between GMV and SDS Score

**Figure 3:**
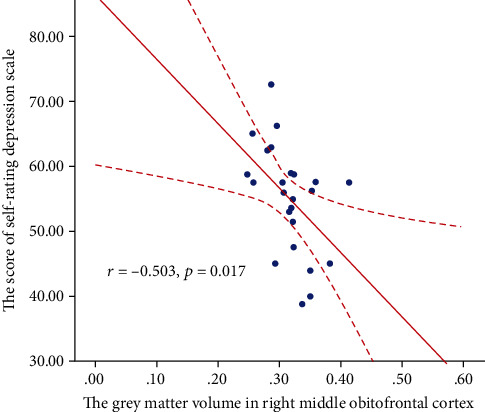
The correlation between grey matter volume (GMV) and clinical variables in spinal cord injury (SCI) patients. The partial correlation analysis revealed a negative correlation between GMV in the right middle orbitofrontal cortex (OFC) and the score of Self-rating Depression Scale (SDS) in SCI patients (*r* = −0.503 and *p* = 0.017).

Partial correlation analysis only showed negative correlation between GMV in the right middle OFC and the SDS score in SCI patients (*r* = −0.503 and *p* = 0.017, Figure 3). However, no significant correlation was found between GMV of the rest ROIs (i.e., left middle frontal cortex, left inferior OFC, right superior OFC, right ACC, left inferior OFC, left middle OFC, and right putamen) and either of the injury duration, injury level, VAS, AIS, ICF scale, SDS score, or SAS score in SCI patients (all *p* > 0.05).

## 4. Discussion

In this study, the VBM and graph theoretical network analyses were used to demonstrate the alterations of GMV and GMV-based SCNs in complete thoracic SCI patients within one postinjury year, and partial correlation analysis was used to explore the association between GMV of structurally changed brain regions and clinical variables. In these patients, we only found GMV reduction in the left middle frontal cortex, left inferior OFC, and right superior OFC, which play an important role in the regulation of psychological or cognitive functions [47–49]. Compared with healthy controls, the GMV-based SCNs in SCI patients showed significant changes of topological properties in regional network evidenced by increased *BC* in the right ACC and left inferior OFC and nodal degree and efficiency in bilateral middle OFCs, as well as decreased *BC* in the right putamen. In addition, the GMV of right middle OFC was negatively associated with score of SDS. These findings may provide new insights into the structural reorganization of brain after SCI at the early stage.

In the present study, the GMV in psychology-cognition-related brain regions including frontal cortex and OFC significantly decreased in SCI patients compared with healthy controls. But majority of previous studies found structural changes in sensorimotor brain regions (M1 [12], S1 [14, 15], and SMA [16]), promoting the development and application of rehabilitation sessions including sensory and motor trainings. The sensory and motor trainings can provide repetitive stimulations to corresponding brain regions, which may potentially prevent or postpone irreversible structural changes in these patients. However, there are also studies [18, 50] reported GMV changes in nonsensorimotor brain regions after SCI, including frontal cortex and OFC, which was consistent with our findings. Frontal cortex plays a critical role in regulation of cognition [49], and structural abnormalities in OFC have also been found in psychological diseases including depression and anxiety [47, 48]. Furthermore, there is close relationship between psychological and cognitive functions [51]. Psychological and cognitive interventions were inadequate or even neglected in many SCI patients. According to the theory of activity-dependent brain reorganization [52], the potential reason why GMV reduction after SCI mainly involved psychology-cognition related rather than sensorimotor brain regions might be that physicians focused more on sensory and motor trainings, and psychological and/or cognitive interventions might be relatively insufficient.

This study also investigated SCI-related alteration of topological properties of global network. The results indicated that structural network segregation and integration in brain were not changed in SCI patient in the first postinjury year. Similar to GMV, the results of regional network analysis showed that SCI also led to significant changes of topological properties of regional structural network in nonsensorimotor brain regions. Nodes with high structural nodal degree and *BC* indicate that nodes are highly interactive with the other nodes in the regional structural network [46]. The present findings indicated that the information transfer through bilateral middle OFCs, right ACC, and left inferior OFC were more efficient in SCI patients than healthy controls, but less efficient in the right putamen. Structural alterations in psychology-related brain region of present study were consistent with clinical findings that almost all of the SCI patients in the present study experienced mild-to-severe depressive feelings. And we did find that greater GMV reduction in psychology-related brain region was related with more severe depressive feelings. Plenty of studies have focused on the alterations of functional network in brain after SCI, instead of structural network [31–33]. However, SCNs were more commonly applied in psychological diseases, and higher *BC* was also noted in the OFC in depression [53]. It is a common knowledge that ACC is activated in pain [54], which was consistent with the present results that almost all of SCI patients experienced mild-to-severe neuropathic pain. Furthermore, the presence and severity of pain may also be related with depressive and anxious feelings [55]. Putamen is one of the important components of the striatum, which is known for motor coordination [56]. The lacking of functional walking and running in complete thoracic SCI patients may have reduced the demand of coordination functions in bilateral lower extremities or between upper and lower extremities.

According to above findings, SCI patients had impairments both in psychological status and alterations in psychology-related brain region. It involves a complex issue that how a nonbrain disease (e.g., SCI) impacts the brain structure. It may potentially be summed up in two aspects. Firstly, the development of psychoneuroimmunology demonstrates that there is a bilateral connection between depression and inflammation [57]. The inflammation associated with depression does not involve a major destruction of the blood-brain barrier (BBB) but primarily activates localized immune mechanisms inducing the activation of central sympathetic nervous system, the hypothalamic-pituitary-adrenal (HPA) axis, and proinflammatory mediators [58]. Secondly, brain-derived neurotrophic factor (BDNF) is a secretory protein in the neurotrophin family and is a crucial mediator of development of axon and pruning of dendrite [59]. It was proved that BDNF was involved in both depression and anxiety [60, 61]. The expression of BDNF was found decreased in dendrites, impairing structural integrity with retraction of distal dendrites of primary cortical neurons and decreased dendritic complexity [62]. Thus, the influence of psychological status on brain structure might be the potential way that SCI induced structural reorganization in brain.

Therefore, SCI not only impairs the physical functions, but also the psychological status in SCI patients. However, current rehabilitation strategies mainly focus on sensory and motor trainings, which might prevent irreversibly structural brain changes in sensorimotor brain regions. Previous studies more focused on functional alteration instead of structural reorganization after SCI, especially in psychology-cognition-related brain regions. However, SCI patients also have an increased risk of psychological problem that anxiety and depression are inevitable consequences after SCI [63–65]. The negative influence of depression and anxiety would not significantly diminish even over 2 years [66]. Furthermore, the cognitive impairment also exists and affects the QoL of SCI patients and their caregivers [51]. The present study emphasized the importance of psychological and cognitive evaluations for SCI patients and neuropsychological interventions including cognitive behavioral therapy (CBT), psychoeducation, motivational interviewing, and interpersonal therapy at the early stage following SCI.

## 5. Conclusion

In summary, SCI has profound effects on psychological status, as well as brain structures including GMV and SCNs within one postinjury year. GMV reduction after SCI mainly involved psychology-cognition-related brain regions rather than sensorimotor brain regions. The efficiency of regional information transmission in psychology-cognition brain regions increased after SCI. Patients may experience depressive and/or anxious feelings at the early stage, and greater GMV reduction in psychology-related brain region was related with more severe depressive feelings. The neuropsychological therapies are suggested to be taken account into the current rehabilitation sessions at the early stage to prevent psychological and cognitive dysfunction as well as irreversible brain structure damage.

## 6. Limitations

The sample size of the present study was relatively small. A cohort with larger samples may be helpful to obtain more information in the future study. The longitudinal comparison of structural changes between pre- and posttherapies needs further investigation. Longer follow-up duration may be worthwhile to investigate time-dependent effects of SCI. The assessment of cognitive function following SCI is also worth exploring in the future.

## Figures and Tables

**Table 1:** Demographic characteristics and clinical variables of the spinal cord injury patients.

No.	Age (years)	Gender	Etiology	Duration (month)	Injury level	AIS	Motor (0-100)	Sensory (0-224)	VAS	ICF scale
1	41	M	Fall injury	6	T8	A	50	125	5	120
2	46	F	Crushing injury	9	T7	A	50	123	5	138
3	40	M	Fall injury	9	T10	A	53	141	6	112
4	52	M	Vehicle accident	5	T10	A	50	140	7	121
5	19	M	Crushing injury	10	T11	A	56	82	6	121
6	43	M	Crushing injury	3	T12	A	50	156	3	78
7	23	M	Fall injury	6	T10	A	52	136	4	100
8	28	M	Fall injury	9	T11	A	53	148	5	105
9	53	M	Fall injury	1	T11	A	50	146	4	100
10	23	M	Crushing injury	10	T9	A	50	132	6	125
11	32	M	Fall injury	3	T10	A	52	141	0	120
12	43	M	Fall injury	6	T11	A	54	148	2	107
13	33	M	Fall injury	3	T11	A	52	148	10	124
14	39	M	Fall injury	4	T3	A	50	86	3	140
15	29	M	Fall injury	6	T11	A	52	149	3	142
16	61	F	Fall injury	1	T7	A	42	112	2	145
17	44	M	Vehicle accident	2	T3	A	50	88	2	129
18	49	M	Fall injury	7	T11	A	50	148	6	76
19	33	M	Vehicle accident	2	T11	A	60	78	3	126
20	50	M	Fall injury	11	T10	A	60	143	8	136
21	34	M	Fall injury	3	T9	A	50	132	3	125
22	43	M	Vehicle accident	10	T6	A	42	108	3	122
23	46	M	Fall injury	4	T10	A	52	136	0	124
24	20	M	Fall injury	4	T6	A	40	100	0	129

Duration refers to the interval between injury and entry to this study. Injury level refers to the neurological level of spinal cord injury. AIS scale rates the severity of spinal cord injury: A, complete—no sensory or motor function is preserved in sacral segments S4–S5; B, incomplete—sensory but not motor function is preserved below the neurological level and extends through sacral segments S4−S5; C, incomplete—motor function is preserved below the neurological level, and more than half of the key muscles below the neurological level have a muscle grade of <3; D, incomplete—motor function is preserved below the neurological level, and at least half of the key muscles below the neurological level have a muscle grade of >3. M: male; F: female; AIS: American Spinal Injury Association Impairment Scale; VAS: visual analog scale; SDS: Self-rating Depression Scale (SDS); SAS: Self-rating Anxiety Scale; ICF: International Classification of Functioning, Disability and Health.

**Table 2:** Intergroup differences of demographic characteristics and clinical variables.

	SCI patients (*n* = 24)	Healthy controls (*n* = 26)	*t/χ* ^2^	*p* value
Age (years), mean (SD)	38.50 (11.19)	38.38 (10.63)	0.037	0.970
SDS, mean (SD)	55.65 (8.74)	47.47 (3.87)	4.392	<0.001
SAS, mean (SD)	48.02 (7.14)	46.41 (4.65)	0.988	0.328
Gender, male no. (%)	22 (92%)	24 (92%)	0.007	0.933

SCI: spinal cord injury; SD: standard deviation; SDS: Self-rating Depression Scale; SAS: Self-rating Anxiety Scale.

**Table 3:** Regions showing significantly decreased grey matter volume (GMV) in spinal cord injury (SCI) patients.

Regions of decreased GMV	Hemisphere side	Cluster size (voxels)	Peak *T* value	Peak MNI coordinate (mm)		
				*x*	*y*	*z*
Middle frontal cortex	L	296	7.762	-33	47	3
Inferior OFC	L	200	5.898	-35	30	-11
Superior OFC	R	126	6.474	30	48	-6

GMV: grey matter volume; OFC: orbitofrontal cortex; L: left; R: right.

**Table 4:** Brain regions are showing significant differences in any of the three nodal characteristics.

Brain region	*p* value		
	BC	Degree	Efficiency
SCI patients>healthy controls			
Right ACC	0.008	-	-
Left inferior OFC	<0.001	-	-
Left middle OFC	-	0.002	0.006
Right middle OFC	-	0.002	0.008
SCI patients<healthy controls			
Right putamen	0.004	-	-

BC: betweenness centrality; SCI: spinal cord injury; ACC: anterior cingulum cortex; OFC: orbitofrontal cortex.

## Data Availability

The data used to support the findings of present study are available from the corresponding authors upon request.

## Supplementary Materials

Supplementary Materials Table S1: intergroup differences of topological properties of global network. Figure S1: changes in topological properties of global network of spinal cord injury patients and healthy controls as a function of increasing network density.
