# Supplementary material for: Altered Topological Properties of Grey Matter Structural Covariance Networks in Complete Thoracic Spinal Cord Injury Patients: A Graph Theoretical Network Analysis
Source: Neural Plast. 2021 Feb 1;2021:8815144. doi: 10.1155/2021/8815144 (PMC7872768; doi:10.1155/2021/8815144)
Supplement: Supplementary Materials — Table S1: intergroup differences of topological properties of global network. Figure S1: changes in topological properties of global network of spinal cord injury patients and healthy controls as a function of increasing network density. [file 8815144.f1.docx]

**Supplementary materials**

**The section of global network analysis as follows:**

**Method**

**Global Network Analysis.** Topological properties of global network including clustering coefficient (*Cp*), characteristic path length (*Lp*), small-worldness index, global efficiency (*Eglob*) and local efficiency (*Eloc*) were used to characterize the global topological organization of structural covariance network (SCN). Briefly, *Cp* of a node is defined as the ratio of number of existing edges to the number of all possible edges in the node’s direct neighbors. *Cp* of network is the mean of *Cp* of node for all ROIs in the brain and is a measure of network segregation. The *Lp* of a network is the mean shortest path length between all pairs of nodes in the network and is a measure of network integration. Small-worldness index means the ratio of normalized *Cp* and normalized *Lp*, $small-worldness index= \frac{\frac{Cp}{{Cp}_{rand}}}{\frac{Lp}{{Lp}_{rand}}}$ , where *Cp_rand_* and *Lp_rand_* are the mean *Cp* and *Lp* of 5000 random networks. A network was considered small-world when the ratio was more than 1. Regional efficiency is defined as the mean of the inverse of the shortest path length between a given node and all of the rest nodes, and *Eglob* is the mean of regional efficiency for all nodes. *Eloc* of a node is defined as the *Eglob* of the subgraph composed of the nearest neighbors of the node, and the *Eloc* of a network is defined as the mean of *Eloc* for all nodes.

**Results**

There was no significant difference in the global network topological properties including *Cp*, *Eglob*, *Eloc*, small-worldness index, and *Lp* between spinal cord injury (SCI) patients and healthy controls (all *p* > 0.05) (Table S1, Figure S1).

**Table S1. Inter-group differences of topological properties of global network**

| Global network properties | *p* values |
| --- | --- |
| Clustering coefficient | 0.412 |
| Global Efficiency | 0.308 |
| Local Efficiency | 0.888 |
| Sigma | 0.601 |
| Characteristic path length | 0.326 |

**
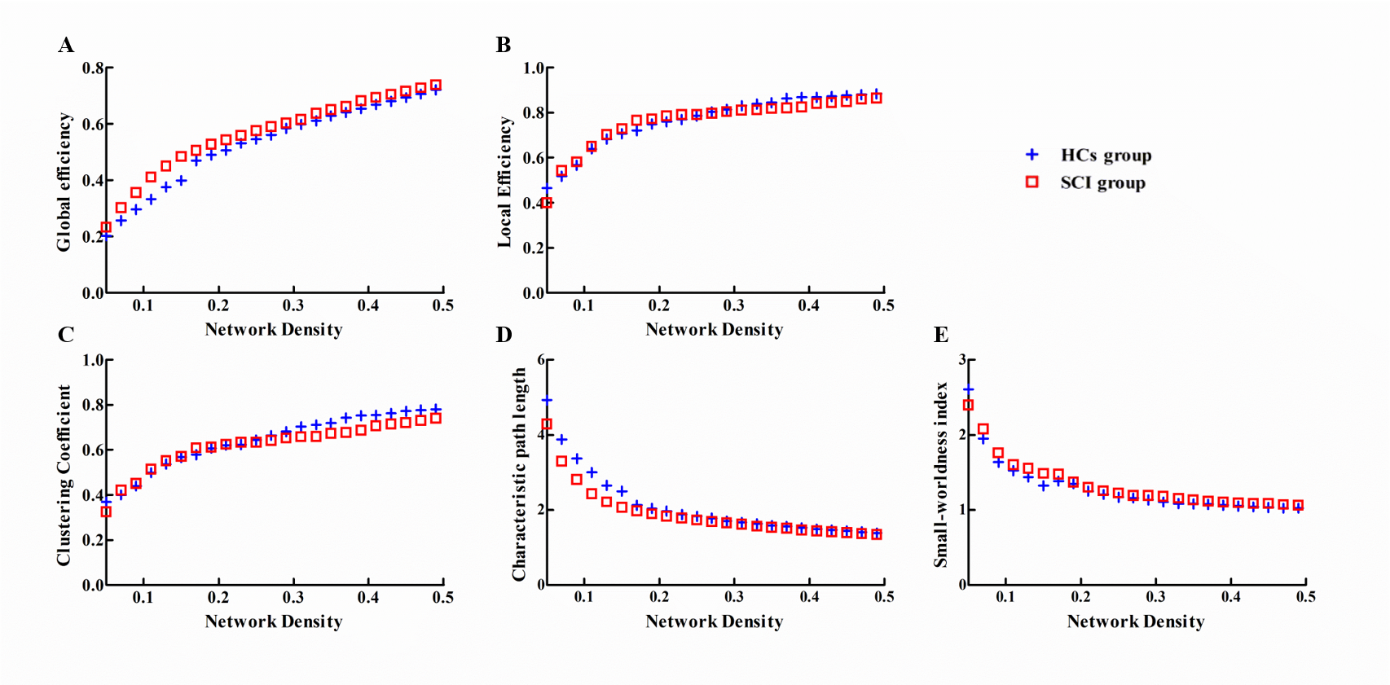
**

**Figure S1. Changes in topological properties of global network of spinal cord injury patients and healthy controls as a function of increasing network density.** (A) global efficiency, (B) local efficiency, (C) clustering coefficient, (D) characteristic path length and (E) small-worldness index of spinal cord injury patients and healthy controls. *SCI, spinal cord injury. HCs, healthy controls.*

**Discussion**

This study also investigated SCI-related alteration of brain global network properties. Both SCI patients and healthy controls showed a small-world property in the structural covariance networks. Despite the presence of a small-world topology in both groups, there was no inter-group difference in other global network topological properties. The results of global network analysis indicated that SCI did not change the combination of structural network segregation and integration.
